# A Review of Analytical Techniques for the Determination and Separation of Silver Ions and Its Nanoparticles

**DOI:** 10.3390/nano13071262

**Published:** 2023-04-03

**Authors:** Miroslav Rievaj, Eva Culková, Damiána Šandorová, Jaroslav Durdiak, Renáta Bellová, Peter Tomčík

**Affiliations:** Electroanalytical Chemistry Laboratory, Department of Chemistry and Physics, Faculty of Education, Catholic University in Ružomberok, Hrabovská Cesta 1, SK-034 01 Ružomberok, Slovakia; eva.culkova@ku.sk (E.C.); damiana.sandorova@ku.sk (D.Š.); jaroslav.durdiak@ku.sk (J.D.); renata.bellova@ku.sk (R.B.)

**Keywords:** silver, silver nanoparticles, silver detection, trace amounts, analytical methods, AgNPs

## Abstract

Many articles have already been published dealing with silver ions and its nanoparticles, but mostly from the environmental and toxicological point of view. This article is a review focused on the various analytical techniques and detection platforms used in the separation and determination of mentioned above species, especially on the trace concentration level. Commonly used are optical methods because of their high sensitivity and easy automation. The separation methods are mainly used for the separation and preconcentration of silver particles. Their combination with other analytical techniques, mainly inductively coupled plasma mass spectrometry (ICP-MS) leads to very low detection limits of analysis. The electrochemical methods are also powerful and perspective mainly because of the fabrication of new sensors designed for silver determination. All methods may be combined with each other to achieve a synergistic improvement of analytical parameters with an impact on sensitivity, selectivity and reliability. The paper comprises a review of all three types of analytical methods on the determination of trace quantities of silver ions and its nanoparticles.

## 1. Introduction

### Silver, Its Compounds and Forms

The extraordinary features of silver are known from ancient times. Silver and its compounds played an important role in industrial and biological fields [1]. Attention is currently being also paid to its toxic properties which are the main threat to the ecosystems and human health [2,3,4].

Silver is an important environmental contaminant in sea, river and industrial waters [5,6]. The silver soluble forms in industrial waters occur as an impurity from copper, zinc and arsenic treatments [7]. The crucial source of silver entering the environment comes from humans; mainly from the pharmaceutical industry and medicine, photography, manufacturing of electronic devices, jewelry, coins and others [5,8].

Silver antibacterial properties are used in the textile industry and in water purification processes [9]. It was found that the cotton treated with the impregnation using silver nano-colloid particles has antibacterial properties showing that over 99% of *Staphylococcus aureus* and *Escherichia coli* cells are killed at a silver content on cotton of approximately 88 mg kg^−1^ [10].

The market consumption of silver and its compounds is higher than 30,000 tons annually in the last thirteen years. The influence of silver species emissions on the environment significantly depends on their speciation.

As mentioned above due to its wide industrial use, silver may enter the environment and adversely affect aquatic ecosystems if the permissible limit announced by the Environmental Protection Agency (EPA) is exceeded. The agency is also pointing out the antagonistic properties of silver to some biologically important species, e.g., copper, selenium and vitamin E [11]. The principle of toxicity to some bacteria and viruses is related to the silver ions inhibition of the mercaptoprotease biological function [12,13,14]. The cell membrane destroying by Ag^+^ biological enrichment may lead to skin irritation and gastric distress or organ edema (swelling) [15,16]. The safe concentration of Ag^+^ in the human body is lower than 0.05 mg kg^−1^ and in drinking water not higher than 0.05 mg L^−1^ [17,18].

Recently, silver nanoparticles (AgNPs) are increasingly used in industry, science (biosensors production) and biomedical applications [19]. This leads during production, storage and application to the release this form into the environment which after physical, chemical and biological transformations increased toxicity and harmful effects of silver [20]. Several studies demonstrated that AgNPs are toxic to the cells of mammals [21]. Exposure to AgNPs occurs by drinking or direct skin contact implying associated health risks. The development of effective analytical methods for the analysis of silver nanoparticles in low concentration levels in the environment and food products is a crucial factor in the prevention of these risks [22]. The characteristic parameters of nanoparticles such as charge, size, as well as aggregation ability in certain media may predict the behavior of nanoparticles. The smaller nanoparticles have a larger surface area and they can be more easily excreted from the organisms [23]. The European Union takes this issue into account by incorporating a new and robust analytical methodology for rapid detection, quantification and characterization of AgNPs using the SERS method [24].

Silver compounds have been used traditionally in the medical field, especially in surgical treatments where the antiviral and antimicrobial properties of silver were correctly documented. These effects depend not only on their concentration but also on size and the method of their preparation.

The current information was discussed recently worldwide to prove the antibacterial and antiviral effects of silver (Ag) and/or AgNPs to fight the COVID-19 pandemic. It was concluded that AgNPs are strongly bound to the SARS-CoV-2 virus eliminating its binding on the host cell. This may lead to virus death [25,26].

The chemically modified AgNPs with citrate in environmental samples and body fluids to simulate their real behavior were investigated. The kinetics of their dissolution in these media may allow us to deeper understand their effects on humans and ecosystems [27,28].

## 2. Analytical Techniques for Silver Determination

### 2.1. Spectrometric Methods

*Colorimetry* is one of the traditional spectrometric analytical methods. Colorimetric methods are generally simple, highly sensitive and may easily use portable detection devices [29,30]. Colorimetric methods are commonly used in the detection of various hazardous ions and contaminants [31]. Recently, due to its high sensitivity and selectivity has gained more interest in analytical determination using gold nanoparticles (AuNPs) [32]. Gold nanoparticles are generally primarily selected for their simple, highly sensitive diagnostic capabilities for fast detection of various analytes. This group of methods has high extinction coefficients with the possibility to visually detect the target species and minimize the use of expensive instruments [33]. Gold nanoparticles produced by a chemical reduction are very well dispersed forming a specific red-colored solution corresponding to surface plasmon resonance absorbance maximum at 520 nm. Due to their easy synthesis, desirable properties, biocompatibility and environmental friendliness, they are suitable for use in many scientific and technological fields [34]. Several reports using gold/silver nanoparticles and have been published [35,36,37,38]. A simple and highly selective colorimetric probe for the determination of Ag^+^ ion was described in paper [37]. The detection system is based on a strong aggregation of AuNPs using ammonium pyrrolidine dithiocarbamate (APDC) as an aggregation agent. This aggregation leads to a red-to-blue color change. If the Ag^+^ ions are present, the mentioned above aggregation is inhibited due to its complex formation with APDC. As Ag^+^ concentration is increased the opposite color change blue-to-red is occurred. This method has a wide dynamic linear range (0.05–0.9 μM) and a relatively low detection limit of 20 nM. This detection system shows high selectivity towards Ag^+^ ions in comparison to other tested metals. This technique was successfully used in the trace analysis of silver ions in real samples of environmental water [37].

**Figure 1:**
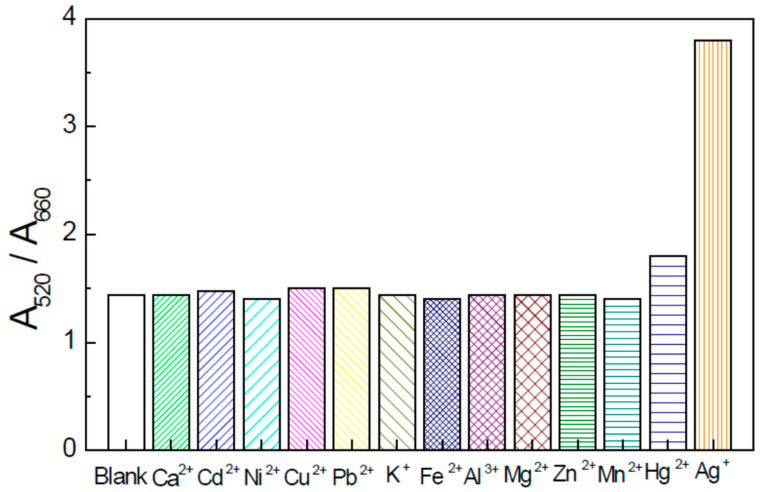
The graphical depiction of the sensor for Ag^+^ ions selectivity and sensitivity compared to other metal ions based on data given in paper [38]. UV-Vis absorption ratio (A520/A660) of gold nanoparticles dispersions mixed with thiamazole in the presence of Ag^+^ with a concentration of 5 nM. The concentration of Pb^2+^, Hg^2+^ and Cu^2+^ was 50 nM. The concentration of other ions was 100 nM (concentration of thiamazole was 2 μM; pH: 7.5).

The selectivity of the Ag^+^ colorimetric determination was investigated in [38]. UV-Vis spectrometry is used to investigate the chemical reaction of various metal ions to thiamazole and their influence on colorimetric probe analytical performance. The estimated average size of the gold nanoparticles during testing was 33 nm. The investigated metal included Cu^2+^, Ca^2+^, Mg^2+^, Cd^2+^, Fe^2+^, K+, Mn^2+^, Al^3+^, Ni^2+^, Hg^2+^, Zn^2+^ and Pb^2+^ were investigated. A graphical representation of the selectivity and sensitivity of the colorimetric probe for Ag^+^ ions compared to other metal ions based on data given in paper [38] is depicted in Figure 1.

As can be seen from Figure 1 the interferences of other ions were negligible at the given concentrations. Despite Cu^2+^ and Pb^2+^ concentrations which were 10-fold higher than Ag^+^ concentration no change in signal was achieved in comparison to the blank solution. The only slightly interfering ion was Hg^2+^. It is attributed to the fact that the oxidation potential of mercuric ions is similar to those of Ag^+^ ions. Nevertheless, this interference could be solved by EDTA. The obtained results indicate that high selectivity of the Ag^+^ determination was reached due to the strong linking of the Ag^+^ ions on amino groups and the sulfhydryl groups oxidation by the analyte. The sensor exhibits a linear dynamic range of 0.002–0.015 μM L^−1^. The limit of detection (LOD) of the designed sensor is equal to 0.046 nM L^−1^. This colorimetric sensor was applied for the Ag^+^ analysis of the river water as real samples [36].

*Surface-Enhanced Raman Scattering (SERS)* method is used in the qualitative analysis of many analytes. It is because of its two advantages. The first one is that each kind of chemical bond has its own vibrational energy as well as Raman spectrum is used for the identification of molecules [39]. The second advantage is that the peak area is related to the concentration of analyzed species. SERS is an ultrasensitive analytical method because it can up to 1000 times amplify a weak Raman signal [40,41]. The SERS method has been used for the determination of illicit drugs [42], sensing and diagnostics of gas molecules in the environment [43] and the detection of alternariol [44]. Recently, the SERS method is important both in scientific and industrial fields [45] as well as in the monitoring of surface plasmons [46,47,48,49,50]. Surface plasmon-promoted reactions are highly dependent on the SERS substrate and the probe molecules [51].

The catalytic transformation of p-aminothiophenol (PATP) to imercaptoazobenzene (DMAB) was interrogated by SERS [52,53]. Silver nanoparticles were used as the substrate. The reaction of 4-aminodiphenyl disulfide (APDS) to DMAB on AgNPs in the presence of laser irradiation was also reported [54,55]. The interferences of various metal species (Cu^2+^, Zn^2+^, Mn^2+^, Co^2+^ and Ni^2+^) on the photochemical transformation of 4-aminothiophenol (4-ATP) to 4,4′-dimercaptoazobenzene (DMAB) was studied using the SERS method. The above-mentioned reaction was performed on the surface of gold nanoparticles and gold nanorods at 632.8 nm and 532 nm laser excitation in the presence and absence of metallic cations [56].

*Fluorescence methods* for the determination of silver nanoparticles came into use with nanotechnology development. Nanomaterials with fluorescence ability have unique advantages allowing designing diverse fluorescence sensors [57]. Quantum dots (QDs) are nanomaterials based on semiconductors that have received significant attention due to their unique features. These are narrow and very symmetric emission spectrum, broad excitation spectrum, higher quantum yield, high selectivity and sensitivity together with excellent stability under the light [58].

Much effort has been devoted to the detection of Ag^+^ by single quantum dots as a fluorescence probe. This probe is based on switch on and switch of the intensity of the fluorescence [59]. These single-intensity sensors are usually difficult to calibrate and often show interferences caused by other metal ions. Therefore expensive, toxic and environmentally unacceptable masking agents are required [60]. Furthermore, the approaches are probably not suitable for single and rapid detection because very long analytical procedures are involved in this case. Many other kinds of fluorescent probes, carbon dots and metal nanoclusters, have been reported to detect silver [61,62,63]. The ratio fluorescence technique based on dual emission and/or multi-emission is able to overcome these disadvantages effectively and thus enable the detection of analytes in complicated samples using the ratios of fluorescence signals at different wavelengths [64]. Additionally, ratio-metric fluorescence probes have improved sensitivity and selectivity because background interferences in samples with complex matrices are diminished [65,66].

The ratio-metric fluorescence method for a cheap and sensitive technique for the detection of Ag^+^ was reported in [67]. NAC (N-acetylcysteine) modified with CdTe (cadmium telluride) of various particle diameters together with dually assembled quantum dots were used to determine Ag^+^ by fluorescence ratio method. The method was found to have a wide linear dynamic range of 0–800 nM L^−1^ with a LOD equal to 7.7 nM L^−1^. This meets WHO and EPA standards and capacitates the method for the trace determination of Ag^+^. In addition, the method is interference-free in the presence of a tenfold concentration of various metal ions and no toxic masking species are needed. This method was successfully used for the determination of Ag^+^ in water real samples. The ratio fluorescence detection technique with dual quantum dots of various excitation wavelengths was also used for real water sample analysis [68]. The obtained recovery of Ag^+^ in different ways was in the interval of 95.5–107.3% with the relative standard deviations in the interval from 0.2 to 2.6% (*n* = 3). The high recovery results and small standard deviations approved this method for the analysis of silver ions water as real samples with a complex matrix.

### 2.2. Separation Methods

Some separation techniques such as turbidity point extraction [69,70] or two-step fractionation as well as cation exchange with CdSe quantum dots [71] as a principle of the separation and subsequent determination of silver nanoparticles have been published recently. Even though the results of both methods are reliable, the analytical operations are time-consuming and some poisonous chemicals are often used. Therefore, the development of a simple and acceptable method is still necessary and challenging.

*Solid phase extraction* is an effective approach used for the separation of metals [72,73], especially for both Ag^+^ and AgNPs [74,75]. It was applied for the separation of thiomalic acid-coated AgNPs using anion resin. Separation is due to the electrostatic interactions of ammonium groups in the resin and the carboxylic groups on the surface of silver nanoparticles; however, the cleavage time was found to be very long (more than 42 h) [74]. The extraction with preconcentration of silver nanoparticles was also evaluated by magnetic Fe_3_O_4_ nanoparticles modified with glutathione and dopamine. This method enables simple monitoring in a flow cell with a neodymium magnet. The high recovery (more than 97%) of enriched silver nanoparticles in tap or fresh water was achieved [75].

Ag^+^ silver ions and silver nanoparticles can be separated in solution on silica aminopropyl oxide (SiAP). This species behaves as a solid adsorbent before its determination by inductively coupled plasma optical emission spectrometry (ICP-OES). Both above-mentioned silver species can be extracted from a binary mixture relatively rapidly in 5 min at optimum pH of 3. The adsorption behavior obeyed the Langmuir isotherm at 298 K. The top adsorption capacity of SiAP for both species was 34.01 and 52.91 mg L^−1^. Metallic silver and silver nanoparticles are selectively desorbed with thiourea. The presented method was found to be easy to use, reliable and economical. It was successfully applied for the determination of silver compounds in consumer products [76].

*Cloud Point Extraction (CPE)* is a relatively novel and ecological method of liquid–liquid extraction. It is a low-cost, convenient, efficient and economical method requiring a short extraction time. Surfactants are non-toxic, non-volatile and non-flammable [77,78]. The cloud point extraction method is commonly used in biology and the environment to isolate hydrophilic and hydrophobic substances [79,80]. It has also a high potential as an analytical method of the preconcentration and extraction of metal cations forming poorly soluble complexes in water. CPE was used for pre-concentration and determination of trace amounts of silver ions in water samples. The calibration curve was linear in the range of 1–500 ng mL^−1^ with a LOD at 0.3 ng mL^−1^ [81].

The cloud point extraction method was also used for the Ag^+^ determination. The pre-concentration of Ag^+^ from the aqueous solution was performed using a nonionic surfactant (Triton X-114) and the chelating agent 6-(4-bromophenylazo) m-anisidine. In the next step, spectrophotometric determination was performed at 514 nm. The efficiency of CPE was found to be affected by several conditions such as Triton X-114 concentration as well as [6-(4-BrPAA)] concentration, pH, time and temperature which need to be optimized. Silver ions react with [6-(4-BrPAA)] to form a complex in a one-to-one ratio in the linear dynamic range of 0.009 to 1.5 μg mL^−1^. The LOD of Ag^+^ ions determination was 5.4 ng mL^−1^ [82].

*Asymmetric Field Flow Fractionation (AF4) and Hydrodynamic Chromatography (HDC)* both usually in conjunction with inductively coupled plasma mass spectrometry (ICP- MS) are used for the separation and determination of inorganic NPs in various samples [83,84]. HDC and AF4 were compared to evaluate their ability to characterize and quantify gold nanoparticles [85]. It was found lower resolution of HDC than AF4. On the contrary, the amount of parent substance retained after completion of the reaction was on average better than that of AF4. It was in the range of 77 to 96% for HDC and 74 to 89% for AF4. Another advantage of HDC in comparison with AF4 is the time of analysis. This time can be reduced to under 10 min compared to AF4′s 30–45 min. In the case of HDC, dissolved substances of low molecular weight are not lost as in the case of AF4 because the ultrafiltration on the membrane is performed in the separation channel. The HDC-ICP-MS provides information about dissolved and particulate forms of metal [86].

Hydrodynamic chromatography is a separation technique similar to liquid chromatography. The analyte in the sample is placed into a tube with beads creating flow channels. The separation of the species is determined by a velocity gradient in the capillaries between beads. The transport of large particles is faster than small ones. The smaller particles spend less time at the edge of the capillaries. Working tubes are of an inert material to minimize non-HDC enthalpic interactions between analytes in the solution and the beads. Non-HDC effects are minimized by salts adding and surfactants in the mobile phase [87]. A series of tubes were used for the separation and determination of the particle diameter of colloidal silica, carbon blacks and polystyrene latexes [88].

HDC in connection with ICP-MS was used in the environmental analysis of nanomaterials [89,90,91]. The determination of the particle size is simple as well as the separation mechanism is also easy [92]. The hyphenation of HDC with ICP-MS in single particle mode (SP-ICP-MS) [93,94] was used to characterize gold nanoparticles in drinking water. Particle mass, hydrodynamic diameter and concentration were successfully determined. HDC-ICP-MS has been successfully used to separate and determine metallic nanoparticles. The ability of HDC-CP-MS to simultaneously determine dissolved forms and nanoparticles of the same element is still a challenging task. Despite limitations, the HDC-ICP-MS method is considered a viable alternative to other methods such as single-particle ICP-MS in which the detection ability towards nanoparticles is significantly influenced by soluble species. The simultaneous detection of ionic and gold and silver in the form of nanoparticles by hydrodynamic chromatography connected with inductively coupled plasma mass spectrometry was investigated in [86]. The addition of 0.05 mM penicillamine to the mobile phase caused very high recoveries for ionic gold and its nanoparticles of 50 nm. In the presence of 1 mM penicillamine in the mobile phase also quantitative recoveries for ionic silver and its nanoparticles up to 40 nm were reached. The best detection limits for gold and silver forms were 0.05 and 0.75 μg L^−1^. This characterization and determination of silver and gold forms were also tested on dietary supplements as real samples. The obtained results are in statistically good agreement in comparison with ones reached by electron microscopy used as an independent technique in this case.

### 2.3. Electroanalytical Methods

**Figure 2:**
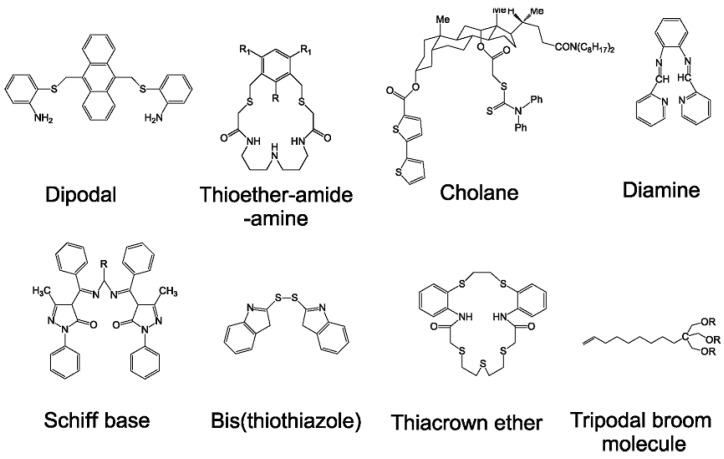
The depiction of the structures of some compounds commonly used as silver ionophores based on data given in paper [1].

*Potentiometry* for the determination of silver is often associated with the development of ion-selective electrodes (ISE). The first silver selective electrode was described in 1968 and it is based on Ag/Ag_2_S crystal membrane [95]. This electrode exhibited a Nernstian dependence of the signal from 0.1 mM to 0.1 M silver ion concentration. It shows a very high selectivity to primary Ag^+^ ions and was only weakly affected (with the exception of Hg^2+^) by other transition metal ions [96]. High selectivity was also important in the fabrication process of liquid membranes for silver ion selective electrodes. The electrode introduced in [97] was of the internally filled type containing dithia-crown ether as silver ionophore. Other ISE methods are based on different types of ionophores. The typical silver ionophore is currently thioether-functionalized calixarene [98]. Electrodes based on thiocarbamate derivatives have specifically controlled Ag^+^ fluxes across the membrane and detection limits in the nanomolar range [99]. This was achieved by various procedures such as tuning the composition of the internal filling solution or membrane conditioning [100]. Detection limits were further improved using plasticizer-free membranes based on methyl methacrylate-decylmethacrylate copolymer with small ion diffusion coefficients for solid-state ISE [101]. A detection limit of 4 × 10^−11^ M was achieved when the conditioning procedure to fluorine membranes modified with perfluorodecylethylthiomethyl benzene as ionophore was used [102]. All reported Ag^+^ selective electrodes have demonstrated that they are suitable for real water sample analysis even on the subnanomolar concentration level. The rather complicated procedure of electrode fabrication and the requirements of the membrane modifications still limit their portability and measurements in dynamic real conditions. The extensive research of ion transfer across various membranes will bring new opportunities into Ag^+^ analysis [103]. Figure 2 shows the structures of some compounds commonly used as silver ionophores depicted on the base of data given in paper [1].

Some of the potentiometric electrodes selective for silver ions which are based on polyvinyl chloride (PVC) membrane and carbon paste electrodes (CPE) have still some disadvantages such as short lifetime or interference of other ions in the analyzed solution. The liquid constitution of PVC also diminishes its use as a concentration detector. Moreover, the relatively large size of CPE prevents their commercialization. Therefore, new sensors with powerful detection parameters are urgently needed for metal ions detection.

Sophisticated screen-printed electrodes (SPE) modified with Alizarin Red S (ARS) for silver ion detection were developed [103]. The content of the ionophore was optimized. The optimization indicates that ink with 30 mg of Alizarin Red S is the best for a monovalent Nernstian slope of 59.95 mV over a considerable concentration dynamic range from 5.0 × 10^−7^ to 1.0 × 10^−2^ mol L^−1^. The detection limit of 5.0 × 10^−7^ mol L^−1^ was reached in this case. The developed sensor has a response time of 7 s and is stable for more than 112 days. During this period no substantial potential drift was noticed. The interaction between silver ions and ionophore modified by ARS on the electrode surface was optically interrogated by scanning electron microscopy (SEM), energy dispersive X-ray analysis (EDX) and FTIR. This sensor has a constant potentiometric response in the range of pH 4–8 and it is thermally stable to 50 °C. The proposed sensor has the ability to selectively distinguish Ag^+^ ions among other common metals and was used for the determination of silver ions in X-ray photographic film. Moreover, it can be used as a working electrode in potentiometric end-point detection of precipitation titrations. 

The comparative study of two different analytical methods, spectrometric and electroanalytical, for the determination of the silver ions and negatively charged silver nanoparticles in an aqueous solution was reported in [104]. The tested AgNP particles have been obtained by electrochemical synthesis (Patent Application EP 18181873) and were highly stable in solution. This has been demonstrated in zeta potential and dynamic laser light scattering measurements. Further UV/Vis spectroscopy and field emission scanning electron microscopy (FE-SEM) were performed for this purpose. Transmission electron microscopy was carried out to find out the average nanoparticle size which was estimated to be 3 nm in diameter at the maximum of the Gaussian distribution curve. The inductively coupled plasma optical emission spectrometry (ICP-OES) was applied for the information of the concentration of silver particles in an aqueous solution. Next, after treatment of the sample solution, and oxidation of the negatively charged silver nanoparticles (AgNPs), the amount of Ag^+^ ions in the solution was determined by Ag^+^ selective electrode. The electrode before the application was adjusted using AgNO_3_ solutions of the concentration range of 1 × 10^−2^–1 × 10^3^ ppm, and the relatively low limit detection of 2.3 × 10^−1^ ppm was reached. In Table 1 are the results of both tested methods [102].

The concentrations achieved by ISE are in statistical agreement with results obtained by ICP-OES. The good correlation between techniques expresses that ISE could be served as a reliable alternative to ICP-OES.

**Figure 3:**
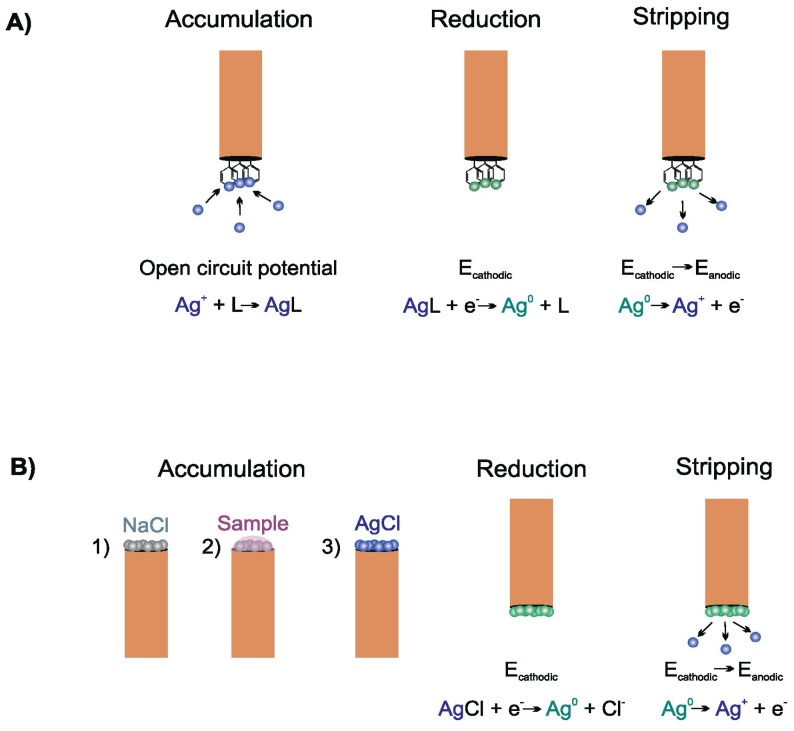
The graphical depiction of two sophisticated procedures for stripping voltammetry trace determination of Ag^+^ ions based on data given in paper [1]. (**A**) Accumulation with ligand silver bond; (**B**) stripping voltammetry using microprobe.

*Voltammetry.* Stripping voltammetry is the most sensitive electroanalytical method for trace analysis of metals. A crucial thing in detection limit lowering to achieve the trace level of silver concentration is an effective optimization of the accumulation step as the most important part of this analytical technique. Several procedures have been used for this purpose [105]. The most common of them is the classical anodic stripping voltammetry (ASV). This sensing platform is generally the most preferred approach for metal and carbon electrodes. A negative deposition potential (typically from −0.7 V to 0 V) is kept for a given deposition time (typically between 60 and 180 s) to reduce Ag^+^ to metallic silver accumulated on the electrode surface as metallic film. Next, this metallic silver is re-oxidized back to silver ion and released from the electrode during this stripping step. The analytical signal is a well-developed oxidation peak. The analytical parameters are strongly influenced by the material of the electrode. The lowest LODs obtained are in the order of nanomoles per liter [106]. More sophisticated procedures for stripping voltammetric trace determination of Ag^+^ ions have been developed. The graphical depictions of two such procedures based on data in [1] are shown in Figure 3.

First of all, the electrode with a proper ligand shows an affinity for silver ions and can be used in the procedure of the accumulation step depicted in Figure 3A. The dipping of the electrode into the measured solution at open circuit potential allows the binding of Ag^+^ which is accumulated directly on the electrode surface. The negative potential reduces accumulated Ag^+^ to metallic silver. By applying an anodic scan of potential the stripping step is started. During this stage, metallic silver is oxidized back to silver ions and diffuses into the bulk phase of the solution. This will appear as an oxidation peak of a better shape in comparison with the reduction peak. Even though the deposition step is relatively longer (3–20 min), this procedure practically always leads to enhanced analytical parameters for subnanomolar concentrations [107]. The choice of an optimal ligand is substantial because its bond with silver ion must be sufficiently strong to allow the accumulation of metallic silver but not so strong to impede its dissolution and release into solution during the stripping step. Since metal–ligand bonds are influenced by pH value, the binding and release of Ag^+^ into solution is reached by careful optimization of storage and stripping solutions. The pH value for each step is different which necessitates the use of two supporting electrolytes. For example, the bis (2-hydroxyacetophenone) butane-2, 3-dihydrazone-modified carbon paste electrode (CPE) requires 0.1 M NaNO_3_ during Ag^+^ accumulation and 5 mM HCl for the dissolution step [108].

In Ref. [1] and in Figure 3B the stripping voltammetric microprobe is introduced. It is based on microelectrode modification with KCl or NaCl followed by poorly soluble silver chloride precipitation when silver ion comes from the bulk phase of the measured solution. The stripping step is similar to the case of silver complexation with ligands. In addition, this constitution of accumulation-stripping experiment allows the analysis of a substantially smaller volume of sample. The low LODs and wide linear concentration range make these sensors an acceptable green mercury-free alternative for silver analysis [109,110,111].

Chemically-modified carbon paste electrodes were widely used in potentiometry for Ag^+^ determination [112,113,114,115,116]. A classic bare carbon paste electrode was introduced for the analysis of Ag^+^ [117]. The lack of selectivity compared to chemically modified carbon paste electrodes was achieved. Several complexing agents for instance alizarin violet [118] or 3-amino-2-mercaptoquinazolin-4(3H)-one [119] have been proposed as carbon paste modifiers for the detection of Ag^+^ ions in the last decade. The complex ligand, 2-hydroxybenzaldehyde benzoylhydrazone (2-HBBH) is often used in the carbon paste electrode as a complexing species for the determination of Ag^+^ in water samples. The arylhydrazone is also an important complexing species. It has excellent donor properties coordinating such a large number of metals [120,121]. Its ability to behave as a polydentate ligand a modifier in carbon paste electrode has not been studied yet.

**Figure 4:**
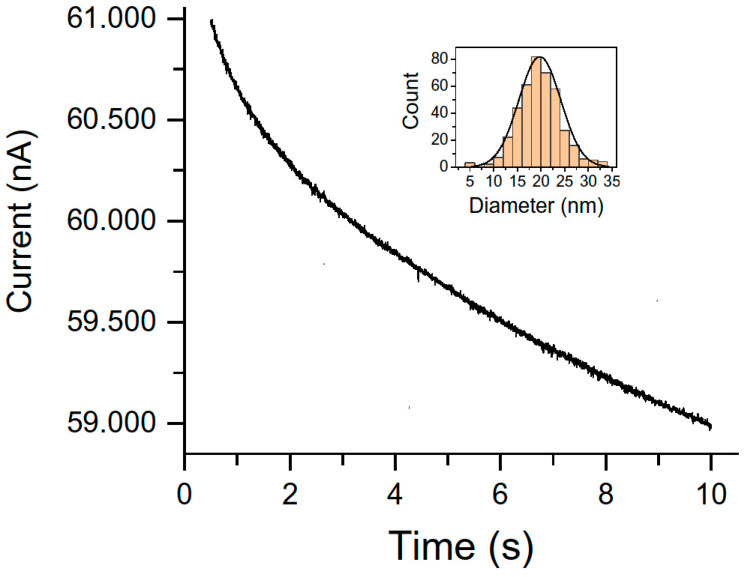
The depiction of a chronoamperogram of a dispersion containing 20 ng L^−1^ silver nanoparticles with a characteristic diameter of 20 nm in 0.02 mol.dm^−3^ NaClO_4_ measured at 0.70 V vs. SCE using carbon fiber microelectrode based on the data given in [24]. Inset: AgNPs size distribution calculated from the spikes measured in the time domain from 2 to 10 s.

*Coulometry.* This electroanalytical technique has also been used to characterize nanoparticles and assess their nanotoxicity. It can provide information about their physical and chemical features as well as size distribution, aggregation and diffusion coefficients or surface oxidation states [122,123]. The electrochemical monitoring of direct and mediated collisions of individual nanoparticles on microelectrodes is based on nanoparticles’ random collisions due to their Brownian motion which makes it possible to measure their sizes. This access is so-called particle collision coulometry (PCC) or nanoparticle impact coulometry as well as anodic particle coulometry. These approaches are reported in [124,125,126]. After impinging on an anodically polarized electrode silver nanoparticles are oxidized and generate low current peaks. The size of these peaks is proportional to the number of atoms taking part in the individual incident of nanoparticle through charge parity thus providing size information. The critical issue is the mathematical treatment of current maxima in data obtained by the nano-impact method to distinguish background from faradaic currents. To solve these problems Fourier transforms or deconvolution of signals originating from nanoparticle aggregation [127] is used. The frequency of recorded impacts is certainly related to the number of nanoparticles, but this method has not been used for quantitative purposes yet [128]. Nano-impact techniques have already been studied from a theoretical point of view. Their real applications are very rare except for the case AgNPs in seawater detection [125]. Determination of extremely low concentrations of nanoparticles is also accessible through measurements of the first arrival time required needed for reaching the electrode surface in random collisions. Femtomolar concentrations of silver nanoparticles have been reported in [129,130]. AgNPs were detected, characterized and quantified by particle collision coulometry (PCC) at a potential of + 0.70 V vs. Ag/AgCl in commercial products [24]. On the base of data given in [24], a typical chronoamperogram for the solution of 20 ng L^−1^ silver nanoparticles in 0.02 M NaClO_4_ as base electrolyte is depicted in Figure 4.

The impact spikes of AgNPs collision events manifested themselves in oxidation peaks with durations of 5–10 ms; the spikes did not appear in blank solutions without nanoparticles. Taking into account the spherical shape of AgNPs, the maximum charge passed in nanoparticle oxidation (1 electron per atom) was calculated according to Faraday’s first law. The diameter of the individual nanoparticle was calculated from the area of above mentioned current–transient peaks.

A typically sized distribution of silver nanoparticles is depicted in the inset of Figure 4. The electrochemical results were compared with data provided by electron microscopy as well as single-particle inductively coupled plasma mass spectrometry. The theoretical and practical singularities of the PCC technique toward the characterization of AgNPs were studied. Reproducible size distributions of the AgNPs were recorded in a diameter range of 10–100 nm. By fitting with power allometric function the relation between the frequency of the nanoparticle collisions on the electrode surface and the concentration of nanoparticles was found. A linear relationship between the number of collisions and the concentration of silver nanoparticles was observed up to 5 × 10^7^ L^−1^. The PCC method was successfully applied for the quantification and size determination of the AgNPs in consumer products containing silver.

Size distributions obtained by the PCC method (of the order 10–20 nm diameters) were in good agreement with the distribution obtained by electron microscopy. The developed PCC method enables the characterization of silver nanoparticle size as well as quantifying their bulk concentration in real samples. A significant amount of Ag^+^ ions should be present, especially in the case of relatively small nanoparticles with diameters of around 10 nm. The SP-ICP-MS method is reliable for larger nanoparticles only with diameters higher than 20–45 nm. However, has good results also for very small diameters, but the fast charge transfer and the complete oxidation of the AgNPs are involved. Statistical analysis showed an agreement between results obtained by TEM/FESEM and PCC at the 95% confidence level in the three studied samples.

## 3. Conclusions

This article is an overview of all three types of analytical methods used for the detection, separation and determination of silver ions and silver nanoparticles, especially at trace concentrations.

Spectrometric methods such as colorimetry, SERS or fluorescence methods are at present based on new nanotechnologies which improve sensor performance, especially in reducing detection limits and increasing selectivity of silver determination. These methods are therefore the most frequently used and their high recovery values (99–104%) proved that they can be used for the determination of different silver forms in real samples.

Separation methods are mainly used for the preconcentration of silver nanoparticles. They are usually combined with another analytical method, for example with ICP-MS. Thanks to SP-ICP-MS methodological approach, the time of analysis was significantly reduced, making determination more convenient and economical.

Electrochemical methods are also powerful and promising. This is due to the development of new sensors which together with better ion flow control provide a better analytical performance of selective electrodes for silver ions making them suitable for its trace determination in the presence of various interfering metallic ions. More attention is gaining the potentiometric sensor in solid form which facilitate the miniaturization of measurements in small and complicated samples. The stripping voltammetric methods have demonstrated applicability in the subnanomolar concentration level of silver ions. The excellent approach is represented by collision and nano impact coulometry allowing the measurement of the size distribution of silver nanoparticles deposited on the electrode surface.

The new approach in all analytical methods will be focused on lowering detector size, its long-term stability, resistance to biofouling and the possibility of portable analysis.

## Figures and Tables

**Table 1:** Ag^+^ concentration in ppm by ICP-OES and ISE analysis, RSD values are in brackets.

Sample	ICP-OES	ISE
1	31.4 ± 1.0 (3.2)	31.8 ± 0.8 (2.5)
2	23.7 ± 0.8 (3.4)	23.4 ± 0.8 (3.4)
3	29.2 ± 0.1 (0.3)	29.3 ± 0.8 (2.7)
4	24.2 ± 0.2 (0.8)	24.1 ± 0.2 (0.8)
5	26.8 ± 0.4 (1.5)	27.2 ± 1.6 (4.0)

## Data Availability

Not applicable.
